# Removal of Antibiotic Resistance Genes from Animal Wastewater by Ecological Treatment Technology Based on Plant Absorption

**DOI:** 10.3390/ijerph20054357

**Published:** 2023-02-28

**Authors:** Han Wang, Delin Du, Yongzhen Ding, Keqiang Zhang, Suli Zhi

**Affiliations:** 1 Agro-Environmental Protection Institute, Ministry of Agriculture and Rural Affairs, Tianjin 300191, China; 2 School of Resources and Environment Northeast Agricultural University, Harbin 150036, China; 3 Key Laboratory of Low-Carbon Green Agriculture in North China, Ministry of Agriculture and Rural Affairs, Beijing 100193, China; 4 China-UK Agro-Environmental Pollution Prevention and Control Joint Research Centre, Tianjin 300191, China

**Keywords:** antibiotic resistance genes, plant absorption, driving factor, removal mechanism

## Abstract

With the aim of controlling the pollution of antibiotic resistance genes (ARGs) in livestock and poultry wastewater, this paper highlights an ecological treatment technology based on plant absorption and comprehensively discusses the removal effect, driving factors, removal mechanism, and distribution characteristics of ARGs in plant tissues. The review shows that ecological treatment technology based on plant absorption has gradually become an important method of wastewater treatment of livestock and poultry breeding and has a good ARG removal effect. In plant treatment ecosystems, microbial community structure is the main driver of ARGs, while mobile genetic elements, other pollutants, and environmental factors also affect the growth and decline of ARGs. The role of plant uptake and adsorption of matrix particles, which provide attachment sites for microorganisms and contaminants, cannot be ignored. The distribution characteristics of ARGs in different plant tissues were clarified and their transfer mechanism was determined. In conclusion, the main driving factors affecting ARGs in the ecological treatment technology of plant absorption should be grasped, and the removal mechanism of ARGs by root adsorption, rhizosphere microorganisms, and root exudates should be deeply explored, which will be the focus of future research.

## 1. Introduction

As the scale and intensification of China’s livestock and poultry farming industry continues to develop, the amount of livestock and poultry manure and wastewater generation is on a rapid growth trend. According to data from the Ministry of Agriculture and Rural Affairs, only in 2015, China produced 2.633 billion tons of wastewater from livestock and poultry farming, but its comprehensive utilization rate was less than 60% [1,2]. If livestock wastewater is not effectively treated and discharged into surrounding water bodies, it will cause environmental pollution in agricultural areas [3,4]. Therefore, exploring efficient and low-cost wastewater treatment technology will be the focus of pollution prevention technology in the livestock and poultry farming industry. In the livestock and poultry industry, veterinary antibiotics are widely used to prevent and treat animal diseases. Their use not only reduces the effectiveness of antibiotics, but can also induce antibiotic resistance genes (ARGs) in animals. Currently, the issue of drug resistance has become a major public health concern worldwide. According to the United Nations Global Environment Outlook, human illness and death due to antibiotic and antimicrobial-resistant infections are expected to be the leading cause of death worldwide by 2050 [5]. In 2019, antibiotic-resistant bacteria and fungi caused more than 2.8 million infections and 35,000 deaths in the United States alone [6]. Currently, a large number of studies have focused on antibiotic resistance gene contamination of livestock and poultry sources. Furthermore, studies related to the removal of antibiotic resistance genes from livestock and poultry wastewater based on ecological treatment techniques of different plant types have been conducted. However, these studies only focused on certain specific conditions and there is a lack of review studies in related fields. Therefore, this paper focuses on plant ecological treatment technology and comprehensively discusses the removal effect, driving factors, and distribution characteristics in plant tissues and the removal mechanism of ARGs, which can provide a theoretical basis for the prevention and control of ARG pollution in livestock and poultry wastewater and help promote the green and sustainable development of plant ecological treatment technology.

## 2. Pollution of Antibiotic Resistance Genes in Livestock and Poultry Wastewater and Its Impact on the Surrounding Environment

### 2.1. Generation of Antibiotic Resistance Genes in Livestock and Poultry Wastewater

According to the Food and Agriculture Organization of the United Nations, in 2020, global production of poultry meat was estimated to be around 134.5 million tons, an increase from the 122.8 million tons produced in 2015. After antibiotics are used in livestock and poultry, on the one hand, it will form selection pressure to make livestock and poultry intestinal microorganisms resistant, thus making livestock and poultry manure carry a large number of ARGs; on the other hand, about 30–90% of antibiotics will be discharged into the environment with livestock and poultry manure, and the antibiotics that enter the environment will not only cause chemical pollution but, most importantly, may induce the production of antibiotic-resistant bacteria (ARB) and ARGs in the environment [7,8,9,10]. As a major livestock farming country, China produces more than 463 million pigs and 106 million cattle annually, accounting for 51.6% and 8% of the world total, respectively [11]. However, China’s livestock and poultry farming is under great pressure due to high emissions, especially the generation of large amounts of wastewater, which put great pressure on the green and healthy development of livestock and poultry farming. In addition, China is also a major antibiotic-using country, with an annual antibiotic use of up to 162,000 tons (about 9 times the total use in the United States), 52% of which are veterinary antibiotics [12].

Therefore, the sources of ARGs in livestock wastewater may have three aspects: (1) livestock wastewater receives ARGs already present in livestock manure; (2) pollutants such as antibiotics and heavy metals in wastewater induce microbial production of ARGs; and (3) proliferation of microbial host bacteria leads to the proliferation of ARGs. Unlike traditional chemical pollutants, ARGs exhibit unique environmental behaviors such as replicability, transmissibility, and environmental persistence due to their inherent biological properties, and ARGs are promoted by mobile genetic elements, such as plasmids, transposons, integrons, insertional sequence common regions, and complex integrons. These ARGs are transmitted between different microorganisms in environmental media through horizontal gene transfer (HGT) mechanisms [13,14] and may enter the food chain and human body through direct or indirect routes, increasing human drug resistance and endangering human public health.

### 2.2. Types and Levels of Contamination with Antibiotic Resistance Genes in Livestock and Poultry Wastewater

Up until now, the presence of antibiotic resistance genes has been detected in different livestock effluents. For example, for cattle farm effluent, Gu et al. [15] showed that tetracycline ARGs (*tet*W, *tet*O, *tet*Q, and *tet*X), sulfonamide ARGs (*sul*1 and *sul*2), streptomycin ARGs (*str*A, *str*B, and *aad*A), and macrolide ARGs (ermB and ermC) were commonly present in cattle farm manure extensively. Ji et al. [16] also showed that *sul*-fa resistance genes (*sul*1, *sul*2, *sul*3, and *sul*A) and tetracycline resistance genes (*tet*W, *tet*O, *tet*M, and *tet*B) were widely distributed in cattle farm manure, with *sul*1, *sul*A, and *tet*W having an abundance of *sul*1, *sul*A, and *tet*W that was relatively high. Antibiotic resistance gene contamination was also prevalent in pig and chicken farm effluent. Mckinney et al. [17] detected sulfonamide ARGs (*sul*1 and *sul*2) and tetracycline ARGs (*tet*O and *tet*W) in oxidation pond sediments from pig and chicken farms, and pig farms had more than chicken farms. Yuan et al. [18] detected tetracycline *tet*A, *sul*1, and *tet*W in effluent from a pig farm in Wuhan. Sulfonamides *sul*1, and quinolones *oqx*B, which are common ARGs, were present in the effluent of a pig farm in Wuhan. Tamminen et al. [19] reported tetracyclines and resistance genes were examined in four livestock farms in Japan where the use of antibiotics had been discontinued, and it was found that in all sampling sites, although tetracycline concentrations were lower than safe-dose concentrations, the copy numbers of *tet*A, *tet*C, *tet*H, and *tet*M genes were significantly higher compared to areas not contaminated by the farms. Not only that, β-lactam ARGs closely related to humans were also commonly detected. For example, *bla*TEM-1, *bla*GES-1, *bla*OXA-1, and *bla*AmpC were detected in 100% of swine farm effluent, with the highest absolute abundance of *bla*TEM-1 and *bla*AmpC, 1.20 × 107 and 1.80 × 107 copies/mL [20]; even some multi-antibiotic resistance genes (*bla*NDM and *mcr*-1) were also commonly detected [21]. This shows that the variety and level of ARGs in the effluent of antibiotic resistance genes from livestock and poultry farming are high, and the pollution situation is very serious.

### 2.3. Impact of Antibiotic Resistance Genes in Wastewater on the Surrounding Environment

**Figure 1:**
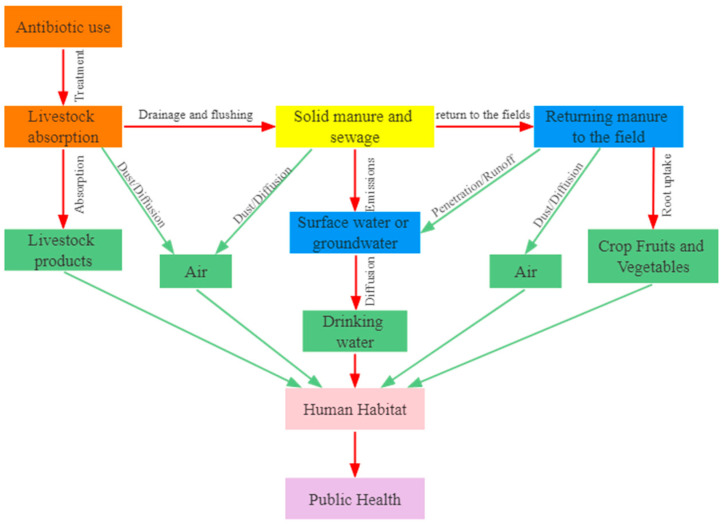
Diffusion of ARGs in the environment from animal wastewater.

At present, China and major developed countries promote the return of livestock manure to the field after disposal. If manure carries a large amount of ARGs, on the one hand, the ARGs in manure and wastewater can enter the soil and crops, on the other hand, the ARGs can enter surface water and groundwater through surface runoff and infiltration. ARGs in both pathways can enter the food chain. The migration and diffusion pathways of ARGs in farms and the surrounding environment are shown in Figure 1.

Numerous studies have shown that ARGs in livestock farm manure can have a significant impact on the surrounding environment. For example, Wu et al. [22] detected 15 tetracycline resistance genes in soil around a pig farm in Beijing, including *tet*A, *tet*C, *tet*E, *tet*G, *tet*K, *tet*L, *tet*A/P, *tet*M, *tet*O, *tet*Q, *tet*S, *tet*T, *tet*W, *tet*B/P, and *tet*X. Lu et al. [23] showed that long-term application of biogas slurry in soils of all sites significantly increased the absolute abundance of total ARG 1.07–1.11 log units and the concentration of tetracycline in soil, as well as increased the abundance of ARGs, transposase genes (*Tn*916/1545), and ARG-related bacteria. Dungan et al. [24] found that dairy farms showed an increase in the abundance and detection of most ARGs (*erm*B, *int*I1, *sul*1, and *tet*M) in soils after wastewater irrigation. Kampouris et al. [25] also showed that the abundance of *sul*1, *qnr*S, *bla*OXA-58, *tet*M, and *intI*1 was significantly higher in soils after wastewater irrigation than in unirrigated soils. Zhu et al. [26] investigated the abundance of *sul*1, *qnr*S, *bla*OXA-58, *tet*M, and *intI*1 among 149 resistance genes detected in manure treatment used in three large suburban pig farms in China, and the 63 resistance genes with the highest abundance were amplified 192~28,000-fold compared to those in soils without manure application. This shows that the manure return process can increase the abundance and detection rate of ARGs in the soil. Therefore, under the strong advocacy of integrated farming and recycling agriculture in China, how to effectively dispose of and reduce the spread of resistance genes or drug resistance in livestock and poultry manure in the face of large areas of manure return to the field is crucial to reduce ecological risks.

## 3. Livestock and Poultry Wastewater Treatment Technology

At present, the main focus of livestock wastewater treatment technology is on biological treatment technology: anaerobic biological treatment and aerobic biological treatment. Although anaerobic treatment technology already has many advantages, there are still some limitations in the removal of nutrients such as N and P, while aerobic treatment technology has greater advantages in the removal of N and P and can slightly compensate for the limitations of the anaerobic process. However, its equipment cost and energy consumption is higher, which creates a huge economic pressure on the farm [27], especially the high concentrations of organic matter, suspended solids, and nitrogen and phosphorus content of livestock effluents, which represent a limitation to the separate application of these two traditional biological technologies. Since both anaerobic and aerobic treatment technologies have their own advantages and disadvantages, combining the two technologies to form a combined anaerobic–aerobic technology is also commonly used in farms. Currently, large farms may have a combined process with multiple treatment units to eliminate pollutants from effluent, but it has been demonstrated that these traditional biological treatment processes are not effective in eliminating ARG pollution. For example, a recent study [28] showed that the absolute abundance of ARGs in cattle farm effluent did not decrease significantly (104–106 copies/mL) after treatment in the collection tank, solid-liquid separation, conditioning tank, and secondary settling tank, suggesting that ARGs in effluent after existing biological treatment processes still pose varying degrees of ecological hazards and potential health risks (probably because microorganisms are vectors of ARGs transmission).Therefore, there is an urgent need to develop a novel, effective, abiotic technology to reduce the level of contamination and risk of secondary transmission of ARGs in cattle farm effluent.

In recent years, ecological treatment technology relying on plant uptake has gradually become an important method of livestock farming wastewater treatment which is increasingly popular. Its main principle relies on the joint action of plant enrichment and uptake, retention of substrate components, and degradation of microorganisms so as to achieve the purpose of deep treatment of wastewater. The technology has the advantages of low cost and easy management and has good application prospects [29]. One example of a large-scale plant-based technology for wastewater treatment is Emscher Park in Germany, which uses constructed wetlands to treat 20,000 cubic meters of wastewater per day. The project has been successful in reducing nutrient and contaminant levels in wastewater and has provided additional benefits, such as recreation opportunities and a wildlife habitat. It is not only effective in removing C, N, and P from wastewater but also has good removal of some emerging pollutants, such as ARGs, antibiotics, and heavy metals [30,31]; more importantly, this technology not only achieves ideal results for pollutant and antibiotic resistance gene removal but also outperforms traditional wastewater treatment systems [32,33,34] and has been used in thousands of large scale systems. It is an ecological treatment technology that is in line with the development of modern green agriculture, being highly efficient and low-cost.

## 4. Plant Ecological Treatment Technology for Livestock Wastewater

### 4.1. Effectiveness of Plant Ecological Treatment Technology on the Removal of Antibiotic Resistance Genes

Plant ecological treatment technology is gradually gaining popularity; therefore, more and more scholars are suggesting to use this technology for decentralized wastewater treatment systems [35]. Up until now, many valuable conclusions have been obtained by different researchers regarding the removal of ARGs (see Table 1). For example, Ávila et al. [32] established two ecological techniques for plant treatment, and the results showed good removal efficiencies for all five target ARGs: 46% to 97% for *sul*1, 33% to 97% for *sul*2, 9% to 99% for *erm*B, 18% to 97% for *qnr*S, and 11% to 98% for *bla*TEM. Chen et al. [36] used an *Cyperus alternifolius* L. constructed ecosystem to treat domestic wastewater, and the results showed that the removal rates of 18 target ARGs ranged from 50.0% to 85.8%. Du et al. [37] used rutabaga to treat pig farm wastewater, and the results showed that the average of *sul*I, *sul*II, *sul*III, *tet*M, *tet*O, and *tet*W removal rates were 67.5%, 85.6%, 95.6%, 87.9%, 97.9%, and 98.5%, respectively. However, the action of plants on ARGs is selective, and ARGs of different mechanisms show different behavioral convergence during the same treatment, while the same ARGs may also show different extinction patterns in ecosystems of different plant types. For example, Chen [38] showed that, after *Cyperus alternifolius* L. treatment, the abundance of *tet*O and *tet*X in wastewater appeared to be enriched with a removal rate of −63.8% and −26.3%, respectively, while all other classes of ARGs showed better removal effects. The reason for this difference could be the different mechanisms of action or the transmission of resistance genes. For example, *tet*M is one of the most common tetracycline ARGs [39] which has been shown to possess the broadest bacterial host range [40], and it is usually associated with chromosomes, conjugates, and transposons of the *Tn*1545-916 family; therefore, *tet*M is ubiquitous in many systems and is widely disseminated in the environment, whereas *tet*O genes are mobile only on binding plasmids [41], which are theoretically less transmissible than *tet*M. Therefore, studying the mechanism of action specific to ARGs in combination with plant physiological properties will not only help enrich the knowledge of ARG removal mechanisms but also contribute to the sustainable development of the whole ecological treatment technology.

### 4.2. Drivers of Resistance Gene Elongation in Plant Ecological Treatment Systems

In terms of microenvironment, there are various factors that influence the behavioral attribution of ARGs during plant treatment of wastewater; microbial communities, mobile genetic elements, environmental factors, and other pollutants are closely related to changes in resistance genes (see Table 2). Indirect driving factors include socioeconomic and environmental factors that influence the use and dissemination of antibiotics and the development of antibiotic resistance. For example, most studies concluded that microorganisms are the host bacteria of ARGs and the growth and reproduction of microorganisms directly affect the changes in the abundance of ARGs [48]. Mobile genetic elements (MGEs) are important indicator elements for the horizontal transfer of ARGs among bacteria, and MGEs are closely related to ARG transmission. Other pollutants (antibiotics, heavy metals, etc.) and environmental factors (TN, TP, TOC, pH, etc.) can directly or indirectly affect the structure of microbial communities in wastewater treatment systems, thus affecting the changes in ARGs [49]. Currently, many studies have focused on the role of different factors in influencing changes in ARGs. For example, Zhu et al. [50] showed that microbial community structure explained 52.3% of the variation in ARGs, while MGEs explained only 7.8%. We recently showed [51] that MGEs within different systems explained most (>50%) of the ARGs, followed by microbial communities. In addition, antibiotic residues can also contribute to the horizontal spread of ARGs [52], but some studies have shown that antibiotics (OTC) have a weak effect on the distribution of ARGs in lettuce tissues, accounting for only 6.3% of the total variance, but significantly correlated with *tet*W, *erm*F, sul1, and intI1 (*p* < 0.05) [49]. Heavy metals (Cu, Zn, Cd, etc.) also induce the production and enrichment of ARGs and have a synergistic induction with antibiotics [53,54]. In turn, other environmental factors can act directly on microorganisms, thus indirectly influencing the dynamic pattern of ARGs [51]. Feng et al. [45] investigated the relationship between soluble organic matter (DOM) and ARG removal and showed that the removal rate of DOM was significantly correlated (*p* < 0.001) with the removal rate of ARGs during the purification of swine farm wastewater by Acorus *calamus*, but the removal rate of *tet*W was not significantly correlated with the removal rate of DOM. Thus, it is evident that determining the driving effect of each factor on ARGs is a hot topic of current research, and the conclusions for the driving effect of each factor on ARGs within different ecosystems vary.

From a macroscopic point of view, process conditions also influence the extinction pattern of ARGs, and, currently, many researchers have examined different process conditions for plant ecological treatment technologies. Direct driving factors conclude wastewater treatment processes such as activated sludge, biological nutrient removal, and membrane bioreactors. Operational parameters such as hydraulic retention time, temperature, and pH can also affect the removal of ARGs and chemical factors. For example, the presence of heavy metals. Moreover, plant type and filler type have a direct effect on ARG removal. Chen et al. [36] compared the removal effect of *Thalia dealbata* Fraser. and *Iris tectorum* Maxim. on 11 ARGs in wastewater through comparative experiments and pointed out that plant type significantly influenced ARG removal. Feng et al. [45] showed that dissolved oxygen has a significant effect on the removal efficiency of the whole plant ecological treatment process; therefore, aeration of the water body is favored by many researchers [30], but some studies have shown that increasing the oxygen capacity does not significantly contribute to the removal of ARGs [37]. The influent method can directly affect the degree of contact between the effluent and the plants and the turbulent flow pattern of the effluent within the system, thus influencing the overall pollutant removal [41,42]. In addition, hydraulic retention time and hydraulic loading are important factors in controlling the removal of pollutants from plant ecosystem effluent, and increasing the hydraulic retention time increases the contact time between pollutants and substrate biofilm, which theoretically contributes to the removal of ARGs [38]; however, excessive hydraulic retention time can lead to an increase in the overall process footprint.

**Figure 2:**
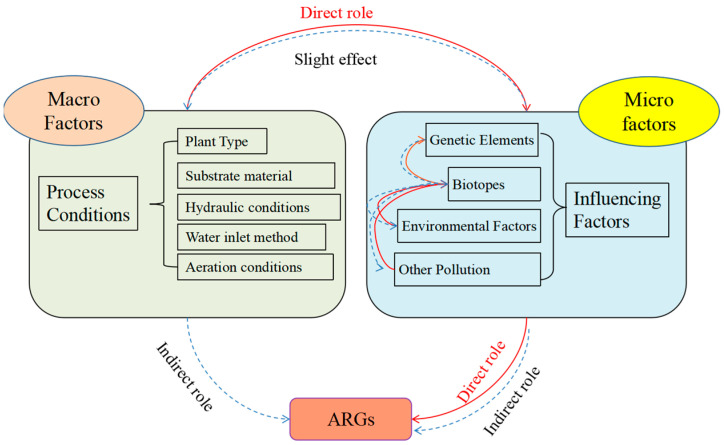
Relationship diagram of different driving factors.

From the above analysis, it can be seen that there are many factors affecting the removal rate of ARGs, and each factor interacts with each other. As shown in Figure 2, all process conditions can be considered as macro factors, and the setting of process conditions directly affects the parameters within the system (considered as micro factors), which theoretically cannot have significant effects on macro factors and therefore can be called “weak effects”. Macro factors ultimately affect ARGs by influencing micro factors. Macro and micro factors such as policy and regulatory frameworks, technological innovations, and funding and investment can also affect the development and implementation of wastewater treatment technologies and the capacity of communities and countries to address the challenge of antibiotic resistance. Therefore, it is not only necessary to clarify the influence of individual factors on ARGs but also to integrate the interactions between various factors in order to find the main factors affecting ARG removal.

### 4.3. Transmission Pathways and Distribution Characteristics of Antibiotic Resistance Genes in Plant Tissues

Throughout the ecological treatment system based on plant uptake, plants play a crucial role in the extinction of ARGs, and the fugitive values of ARGs within different plant tissues determine the risk and probability of ARG transmission to the next level of the food chain. The distribution characteristics of ARGs within plant tissues are a hot topic of research within the ecological transformation system. Notably, many studies have shown that ARGs can be distributed in plant tissues such as roots, stems, and leaves. For example, Yang et al. [56] showed that the plant tissues of celery, cabbage, and cucumber contained culturable bacteria resistant to cefadroxil after cefadroxil selection pressure was applied to the plant growth environment in various tissue sites of plants. The size order was soil samples > leaf peripheral samples > root endophyte samples > leaf endophyte samples. However, not all ARGs can migrate through the plant root system to all tissues of the plant. For example, Duan et al. [49] showed that *sul*1, *sul*2, *erm*F, and *erm*X can migrate from the root endophyte to the leaf part of lettuce, but tetracycline ARGs were very low in the leaf part, where *tet*W was not detected in the stem and leaf tissues. However, Ye et al. [57] showed that sulfonamide resistant bacteria or resistance genes (*sul*1 and *sul*2) were not detected in new lettuce leaf tissues, while they were detected in old leaf tissues (10–7 to 10–9 copies/16S copies). This shows that ARGs are unevenly distributed in different parts of the plant; moreover, different species of ARGs have different distribution characteristics.

### 4.4. Mechanism of Removal of Antibiotic Resistance Genes

**Figure 3:**
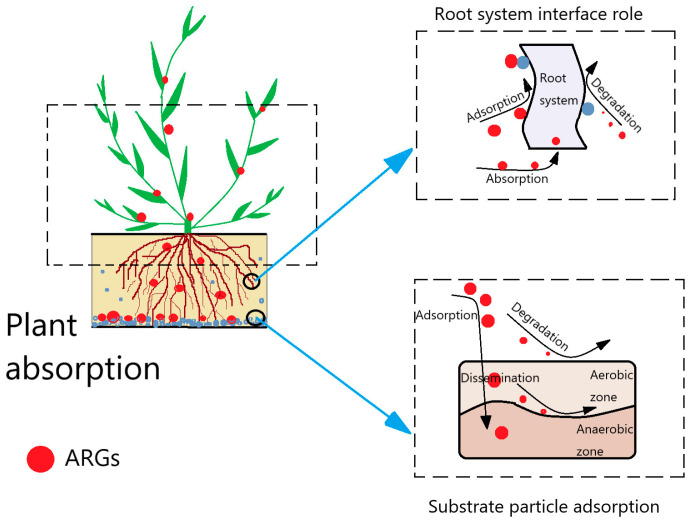
Schematic diagram of the removal mechanism of ARGs.

ARGs, as an emerging pollutant, exhibit a different behavior and fate in different plant ecological treatment systems in terms of species and abundance. A schematic diagram about the removal mechanism of ARGs within the whole plant ecological treatment system is shown in Figure 3. Overall, the removal pathways of ARGs in plant ecological treatment systems include the following three aspects: (1) Biological role: Microorganisms play an important and complex role in ARG removal because they are not only related to the propagation and proliferation of ARGs but may also play a role in degrading ARGs [58,59]. Chen et al. [36] showed that the process of reflectant domestic wastewater microorganisms play a major role in ARG removal (73.7–95.2%). (2) Substrate sorption: Substrate sorption also plays an important role in ARG removal, and the abundance of ARGs in substrate materials showed accumulation in different plant treatment processes, which indicates that substrate materials can sorb ARGs from wastewater to achieve ARG removal [36,43]. Chen et al. [30] clearly pointed out that substrate sorption and microbial degradation are the two main mechanisms of action for ARG removal. (3) Plant uptake: Plant uptake is also an aspect of ARG removal that cannot be neglected. Studies have shown that plant tissues are not completely immune to ARGs and plant root endophytes can acquire some ARGs from root surface stomata and mechanical damage and spread them with plant endophytes so that ARGs reach the stems and leaves [60]. Although some studies have shown that microbial degradation plays a relatively large role in ARG removal while substrate sorption and plant uptake play a relatively small role [36], the role of the latter two is inextricably linked to microbial degradation, and the substrate and plant root system can provide attachment sites for pollutants (ARGs) and microorganisms, thus allowing the microorganisms to more fully contact the pollutants and achieve a better degradation effect [36]. In particular, inter-root microorganisms specific to plant roots may also be important for the removal of ARGs [61,62]. Plant root surface tissue secretions, which regulate root surface pH and redox conditions, provide suitable growth conditions for interfacial microorganisms and also increase microbial activity, thus enhancing the overall biodegradation process.

The question of how endophytic bacteria acquire ARGs and how ARGs spread between plant tissues has been a difficult research problem. Many studies have shown that there is a large overlap between the endophytic colonies of plant tissues and the microbial communities in the peripheral environment of the root system [50,63,64], which indicates that the microbial community composition in plants (especially in the root tissue) is largely influenced by the external environmental microbial community; however, for parts such as leaves that are far from the root tissue, their microbial communities are more differentiated from those of root endophytes. For example, Zhang et al. [65] showed that only 12 bacterial OTUs (Group II) in the root microbial community may spread further into leaf endophytes, indicating differences with the microbial communities of leaf endophytes. In addition, this ability of microbial transmission directly affects the probability of ARG transmission. For example, Duan et al. [49] showed that the thick-walled phylum Firmicutes, a potential host bacterium for ARGs, is unable to migrate from the root system to the stem and leaf tissues, which may be the main reason why *tet*W cannot be migrated to stem and leaf parts. Other studies have also shown that the external environment can serve as a seed bank for microbial communities within the root system and that plant endophytes are mostly acquired horizontally from the external environment rather than being spread vertically from the parent plant via seeds or pollen [66,67]. Therefore, it is important to clarify whether ARGs migrate from effluent to the root system of duckweed. If such a migration exists, do ARGs migrate between the different tissues of duckweed? If so, how do they migrate and spread? Clarifying these questions is the bottleneck to control the spread of ARGs to the next level of the food chain and will help to provide a reference basis for controlling the secondary spread of ARGs and assessing the risk of ARGs entering the food chain.

### 4.5. Conclusions and Outlook

Ecological treatment technology based on plant uptake has become an important method of resource-based treatment of livestock wastewater, which is an environmentally friendly, low-energy, and high-efficiency treatment technology. It has better ecological benefits and landscape functions. The research on the removal of ARGs by plant ecological treatment technology has been very comprehensive and in-depth; however, the question remains as to how to take into account the mechanisms of action of the different types of ARGs and synthesizers. Nevertheless, it is still controversial to improve the removal efficiency of all ARGs. Therefore, this paper summarizes and gives outlook from the following three aspects.

(1) Continue to clarify the key drivers of ARGs. Since ARG removal is influenced by many factors and there are interactions among the various factors, it is a primary task to sort out the relationships among them, which can help to capture the main factors influencing ARGs. In addition, the current research tools mainly focus on comparative experiments and correlation coefficient analysis methods, but these tools have certain limitations. For example, the correlation coefficient analysis method can only analyze two variables and fluctuates greatly due to the number of data sets, while it is difficult to ensure that other factors remain unchanged under the condition that only one factor can be changed at a time for comparative experiments. Therefore, it is necessary to find a technical tool that can sort out the relationship between various factors, including the direct effect between factors and the indirect effect through other factors.
(2) In-depth exploration of the removal mechanism of ARGs. There are various pathways for ARG removal. Although some studies suggest that microbial degradation plays a major role in ARG removal, the role of plant uptake and substrate particle adsorption cannot be ignored. They provide attachment sites for microorganisms and contaminants. In particular, the root interface is a complex mechanism for ARG removal; therefore, the root interface should be the focus of future research. Therefore, the removal mechanisms of ARGs at the root interface should be studied in depth.
(3) Investigate the distribution characteristics and propagation mechanisms of ARGs in plant tissues. After absorbing nutrients from wastewater, most plants recycle them for resource use. The distribution and propagation of ARGs in plants is the key to whether ARGs can enter the next level of the food chain, and there are few studies focusing on this aspect. Therefore, the distribution characteristics of ARGs in different plant tissues and the mechanisms of their transfer should be further clarified in order to assess the ecological risk posed by ARGs in plant ecological treatment systems.

## Figures and Tables

**Table 1:** ARG removal in different ecological treatment processes.

Wastewater Types	Botany Types	Variable Factors	Target ARGs	Removal Effects	References
Domestic wastewater	*Cyperus alternifolius* L.	Artificial aeration and mixing design	*sul*1, *sul*2, *tet*G, *tet*O, *erm*B, *qnr*S, *qnr*D, *cml*A and *flo*R	87.8~99.1%	[30]
Domestic wastewater	*Thalia dealbata* Fraser. and *Iris tectorum* Maxim.	Flow patterns and plant types	*sul*1, *sul*2, *sul*3, *tet*G, *tet*M, *tet*O, *tet*X, *erm*B, *erm*C, *cml*A and *flo*R	63.9~84.0%	[36]
Domestic wastewater	*Cyperus alternifolius* L.	Substrate and hydraulic load	*sul*1, *sul*2, *sul*3, *tet*G, *tet*M, *tet*O, *tet*X, *erm*B, *erm*C, *qnr*B, *qnr*D, *qnr*S, *cml*A, *fex*A, *fex*B, *flo*R, *intl*1 and *intl*2	50.0~85.8%	[38]
Pig farm wastewater	P. *australis*	Vertical Flow Artificial Wetland	*sul*1, *sul*2 and *sul*3	89%, 88% and 84%	[42]
Pig farm wastewater	Hybrid *pennisetum*	Filler type	*tet*M, *tet*O and *tet*W	50%	[43]
Pig farm wastewater	*Arundo donax*	Filler type	*sul*I, *sul*II, *sul*III, *tet*M, *tet*O and *tet*W	67.5%, 85.6%, 95.6%, 87.9%, 97.9% and 98.5%	[37]
Synthetic pig farm wastewater	P. *australis*	Water flow method	*sul*I, *sul*II, *tet*M, *tet*W and *tet*O	99.9% (Sulfonamides); 99.9% (Tetracycline)	[41]
Livestock wastewater	P. *australis*	Exogenous antibiotics and resistant bacteria	73 ARGs	>60%	[44]
Pig farm wastewater after digestion	*Iris pseudacorus*	With or without aeration	*tet*A, *tet*M, *tet*O and *tet*W	87.88%	[45]
Urban wastewater	P. *australis*	Operating conditions	*intI*1, *qnr*S, *sul*1, *sul*2, *bla*TEM and *erm*B	−7.67~92.9%	[32]
Wetlands wastewater	P. *australis*	With or without aeration	*sul*1, *sul*2, *tet*A, *tet*C, *erm*B and *intl*1	12.3~39.2%	[46]
Pig farm wastewater	*Pontederia cordata* and *M. verticillatum* L.	Water flow method	*sul*3, *intI*1, *sul*2, *sul*1, *tet*O, *erm*B, *intI*2, *tet*B/P, *erm*C, *tet*M and *tet*X	87~99%	[47]

**Table 2:** ARG driving factors during different ecological treatment processes.

Wastewater Types	Botany Types	Analysis Method	Target ARGs	Influencing Factors and Conclusions	References
Domestic wastewater	*Cyperus alternifolius* L.	Correlation factor method	*sul*1, *sul*2, *tet*G, *tet*O, *erm*B, *qnr*S, *qnr*D, *cml*A and *flo*R	Dissolved oxygen, antibiotic levels significantly affect microorganisms and thus ARGs	[30]
Domestic wastewater	*Thalia dealbata* Fraser. and *Iris tectorum* Maxim.	Comparison test	*sul*1, *sul*2, *sul*3, *tet*G, *tet*M, *tet*O, *tet*X, *erm*B, *erm*C, *cml*A and *flo*R	Plant type had a significant effect	[36]
Domestic wastewater	*Cyperus alternifolius* L.	Analyzing Data	*sul*1, *sul*2, *sul*3, *tet*G, *tet*M, *tet*O, *tet*X, *erm*B, *erm*C, *qnr*B, *qnr*D, *qnr*S, *cml*A, *fex*A, *fex*B, *flo*R, *intl*1 and *intl*2	Microbial activity is significantly correlated with pollutant removal	[38]
Pig farm wastewater	*Arundo donax*	Correlation coefficient	*sul*I, *sul*II, *sul*III, *tet*M, *tet*O and *tet*W	The removal rate of ARGs was significantly and negatively correlated with the absolute abundance of 16S and ARGs but not with the relative abundance of ARGs	[37]
Synthetic pig farm wastewater	P. *australis*	Comparison test	*sul*I, *sul*II, *tet*M, *tet*O and *tet*W	pH 7-8 is optimal, added oxygen content does not contribute to the abatement of ARGs, and the effect of antibiotics is not significant	[41,55]
Livestock wastewater	P. *australis*	Comparison test	73 target ARGs	Abundance of ARGs promoted by oxytetracycline and exogenous drug-resistant bacteria	[44]
Pig farm wastewater after digestion	*Iris pseudacorus*	Correlation coefficient method	*tet*A, *tet*M, *tet*O and *tet*W	Soluble organic matter composition and content, COD were significantly correlated with *tet*A, *tet*M, *tet*O and not with *tet*W; oxygen content	[45]

## Data Availability

Data is available only when requested from the corresponding author.
